# Toll-like receptor expression and function differ between splenic marginal zone B cell lymphoma and splenic diffuse red pulp B cell lymphoma

**DOI:** 10.18632/oncotarget.25283

**Published:** 2018-05-04

**Authors:** Aurélie Verney, Alexandra Traverse-Glehen, Evelyne Callet-Bauchu, Laurent Jallades, Jean-Pierre Magaud, Gilles Salles, Laurent Genestier, Lucile Baseggio

**Affiliations:** ^1^ Université de Lyon, Université Claude Bernard Lyon 1, INSERM 1052, CNRS 5286, Centre Léon Bérard, Cancer Research Center of Lyon, Lyon, France; ^2^ Service d’Anatomie-pathologique, Centre Hospitalier Lyon-Sud/Hospices Civils de Lyon, Pierre-Bénite, France; ^3^ Laboratoire d’Hématologie Cellulaire, Centre Hospitalier Lyon-Sud/Hospices Civils de Lyon, Pierre-Bénite, France; ^4^ Service d’Hématologie, Centre Hospitalier Lyon-Sud/Hospices Civils de Lyon, Pierre-Bénite, France

**Keywords:** splenic marginal zone lymphoma, splenic diffuse red pulp lymphoma, toll-like receptor

## Abstract

In splenic marginal zone lymphoma (SMZL), specific and functional Toll-like Receptor (TLR) patterns have been recently described, suggesting their involvement in tumoral proliferation. Splenic diffuse red pulp lymphoma with villous lymphocytes (SDRPL) is close to but distinct from SMZL, justifying here the comparison of TLR patterns and functionality in both entities.

Distinct TLR profiles were observed in both lymphoma subtypes. SDRPL B cells showed higher expression of TLR7 and to a lesser degree TLR9, in comparison to SMZL B cells. In both entities, TLR7 and TLR9 pathways appeared functional, as shown by IL-6 production upon TLR7 and TLR9 agonists stimulations. Interestingly, circulating SDRPL, but not SMZL B cells, constitutively expressed CD86. In addition, stimulation with both TLR7 and TLR9 agonists significantly increased CD80 expression in circulating SDRPL but not SMZL B cells. Finally, TLR7 and TLR9 stimulations had no impact on proliferation and apoptosis of SMZL or SDRPL B cells.

In conclusion, SMZL and SDRPL may derive from different splenic memory B cells with specific immunological features that can be used as diagnosis markers in the peripheral blood.

## INTRODUCTION

Toll-like receptors (TLR) recognize a set of different pathogen-associated molecular patterns derived from viruses, bacteria and fungi [1], as well as from various endogenous molecules and auto-antigens [2]. TLR bridge innate and adaptive immune responses by acting as costimulatory signals for B cells and by inducing maturation, proliferation and antibody production [3, 4]. Furthermore, memory B cells seem to acquire the capacity to respond to specific TLR agonists, such as TLR7 and TLR9 [5, 6]. In addition, abnormal TLR expression and/or signaling may play an important role in the pathogenesis of lymphomas, especially in splenic marginal zone lymphoma (SMZL), since TLR pathways are recurrently targeted by genetic changes in this entity [7]. Likewise, molecular lesions of signaling pathways have been discovered in chronic B cell lymphoproliferative disorders by next generation sequencing (NGS) technology. In addition to TLR, these included B cell receptor (BCR), NOTCH, nuclear factor-κB (NF-κB) and mitogen activated protein kinase (MAPK) signaling pathways [8]. Recently, evidence of BCR/TLR interactions has been demonstrated in Chronic Lymphocytic Leukemia (CLL), since simultaneous engagement of BCR/TLR leads to different responses in CLL depending on the mutational status of the BCR [9]. To date, TLR expression has been mainly described in lymphoid malignancies at mRNA level rather than at protein level, and more frequently in follicular lymphoma, CLL and diffuse large B cell lymphoma than in marginal zone lymphoma [10, 11]. More recently, Fonte *et al.* have reported functional TLR (TLR1/2, TLR2/6, TLR9) in neoplastic SMZL B cells, suggesting their role in lymphomagenesis, by promoting the expansion of the neoplastic clone [12].

Among the splenic B cell lymphomas, the splenic diffuse red pulp lymphoma with villous lymphocytes (SDRPL) has been identified as an entity close to but distinct from SMZL [13]. Indeed, each entity presents different clinical, morphologic, immunologic, genetic and molecular features with some overlapping [14–16]. Both entities account for less than 1% of B cell non Hodgkin's lymphoma, and numerous questions remain concerning their cellular origin and lymphomagenesis. Moreover molecular and clinical findings indicate that antigens expressed by common pathogens and specific antigen receptors may be involved in the initiation of selection and stimulation of tumoral B cells [17, 18], implicating the TLR pathway. This study focused on the definition of TLR profile and function in neoplastic B cells from SDRPL in comparison to those from SMZL.

## RESULTS

### TLR profile differs between SMZL and SDRPL

**Figure 1:**
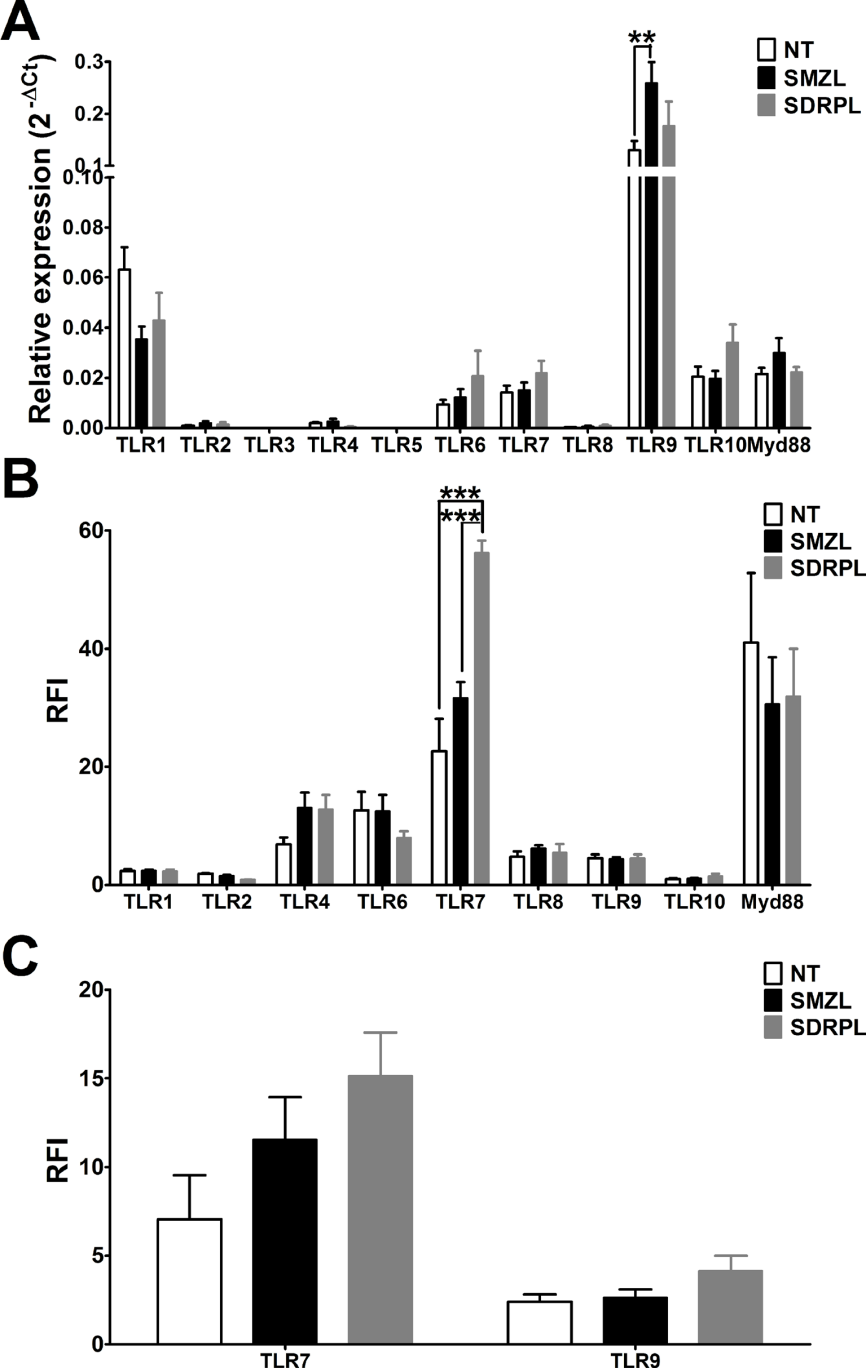
Pattern of TLR mRNA and protein expressions (**A**) mRNA expression and (**B**) protein expression of the different TLR in B cells from non-tumoral, SMZL and SDRPL samples, all from spleen. The relative expression levels of mRNAs were expressed as mean ± SEM. The protein results were expressed as the ratio of fluorescence intensity (RFI), which corresponds to the normalized mean fluorescence intensity (MFI) over the MFI of the isotype negative controls (^**^p < 0.01 and ^***^p < 0.001 with two-way Anova followed by Bonferroni test). (**C**) Expression of different TLR (expressed as RFI) in CD19^+^ B cells from non-tumoral, SMZL and SDRPL samples, all from PB, were represented as mean ± SEM.

B cells from non-tumoral, SMZL and SDRPL samples from spleen, were purified by magnetic cell sorting (purity >95%) and TLR mRNAs expression was quantified by real-time RT-PCR. All TLR mRNAs, except TLR3 and TLR5, were expressed in normal or tumoral B cells from spleen samples. Nearly detectable mRNA levels were found for TLR2, TLR4, TLR8, whereas the highest level detected was for TLR9 (Figure 1A). TLR9 was the only TLR significantly differentially expressed between these different entities, with a higher expression in SMZL (*p* < 0.01).

Subsequently, we performed flow cytometry (FCM) analysis on normal and tumoral spleen samples by gating on CD19^+^ B cells. Since TLR3 and TLR5 mRNA levels were undetectable, their protein expression was not studied by FCM. Intriguingly, there was no direct correlation between the TLR mRNA and protein levels. Low expression of TLR1, TLR2 and TLR10 (Ratio of Fluorescence Intensity, RFI<5), intermediate expression (RFI between 5 and 20) of TLR4, TLR6, TLR8 and TLR9, and very high expression (RFI>20) of TLR7 (Figure 1B) was observed. This contradicting result may be caused by low stability of the specific mRNA as well as translation and/or post-translational modifications of the protein [19, 20]. Interestingly splenic SDRPL B cells presented a distinct TLR profile with significantly higher TLR7 expression (*p* < 0.001), and a trend towards lower TLR2 and TLR6 expression in comparison to splenic SMZL B cells (Figure 1B).

Even if both lymphomas are of splenic origin, circulating tumoral cells are frequent in the peripheral blood (PB). We therefore evaluated the expression of the two significantly expressed TLR, TLR7 and TLR9 protein by FCM on circulating B cells. Expression profile of TLR7 and TLR9 was similar in PB as compared with spleen in both entities as TLR7 expression was higher in B cells from SDRPL than SMZL, and TLR9 was not differentially expressed between SDRPL and SMZL (Figure 1C).

### Expression of IL-6 upon TLR7 and TLR9 agonist stimulations differs between SMZL and SDRPL

Since TLR7 was the most differently expressed TLR between SMZL and SDRPL, and since it shares the same signaling pathway as TLR9 (described by *Fonte et al.* [12] as having functional impact on SMZL B cells), we focused our attention on the impact of TLR7 and TLR9 agonists on these two different lymphoma entities. The functional studies were performed on sorted tumoral B cells using TLR7- and TLR9-specific agonists, Imiquimod (IMQ) and CpG ODN, respectively. Functional signaling of TLR7 and TLR9 was assessed by measuring IL-6 concentration in the culture supernatants after 24 hours of stimulation with TLR7 and TLR9 agonists in circulating B cell samples.

**Figure 2:**
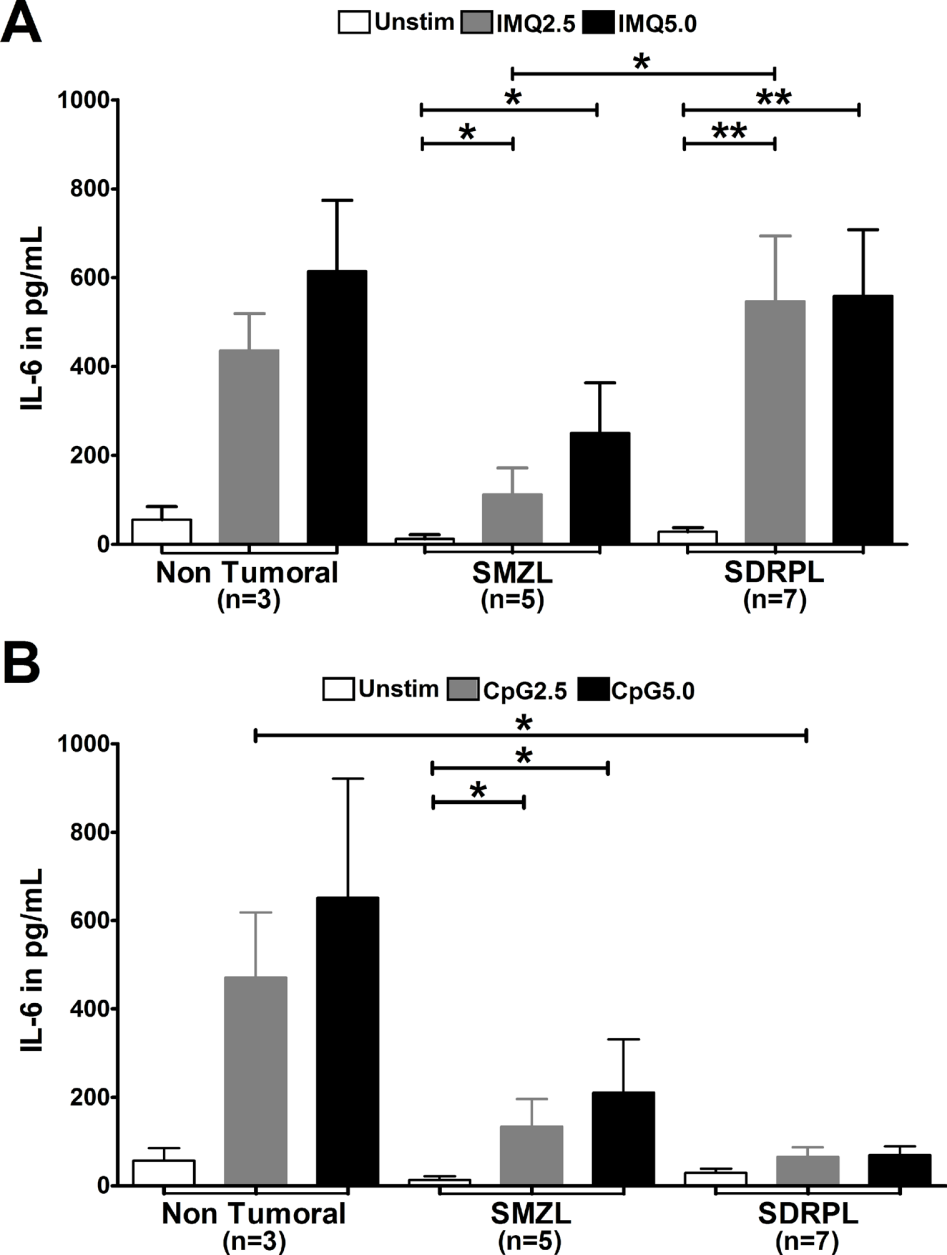
IL-6 production after TLR7 and TLR9 stimulations TLR7 and TLR9 stimulations were both assessed with 2 concentrations (2.5 and 5 μg/mL) of agonists. Concentration of IL-6 was measured in the cell culture supernatants after 24 hours of stimulation, by ELISA. (**A**) Stimulation with TLR7 agonist (IMQ) on PB B cells induced IL-6 secretion in non-tumoral B cells, circulating SMZL B cells and SDRPL B cells. (**B**) Stimulation with TLR9 agonist (CpG) on PB B cells induced IL-6 secretion in non-tumoral B cells and SMZL B cells, no significant secretion was observed in circulating SDRPL B cells. (^*^p < 0.05 and ^**^p < 0.01 with Mann-Whitney *t*-test).

Stimulation with the TLR7 agonist induced IL-6 secretion by both SMZL and SDRPL B cells (Figure 2A, *p* < 0.05, *p* < 0.01, respectively). In addition, SDRPL B cells produced higher IL-6 levels than SMZL B cells in response to TLR7 stimulation (Figure 2A, *p* < 0.05). By contrast, the TLR9 agonist induced a significant IL-6 secretion in SMZL B cells (*p* < 0.05), but not in SDRPL B cells (Figure 2B).

### TLR7 and TLR9 stimulations have no impact on proliferation and apoptosis of SMZL and SDRPL

**Figure 3:**
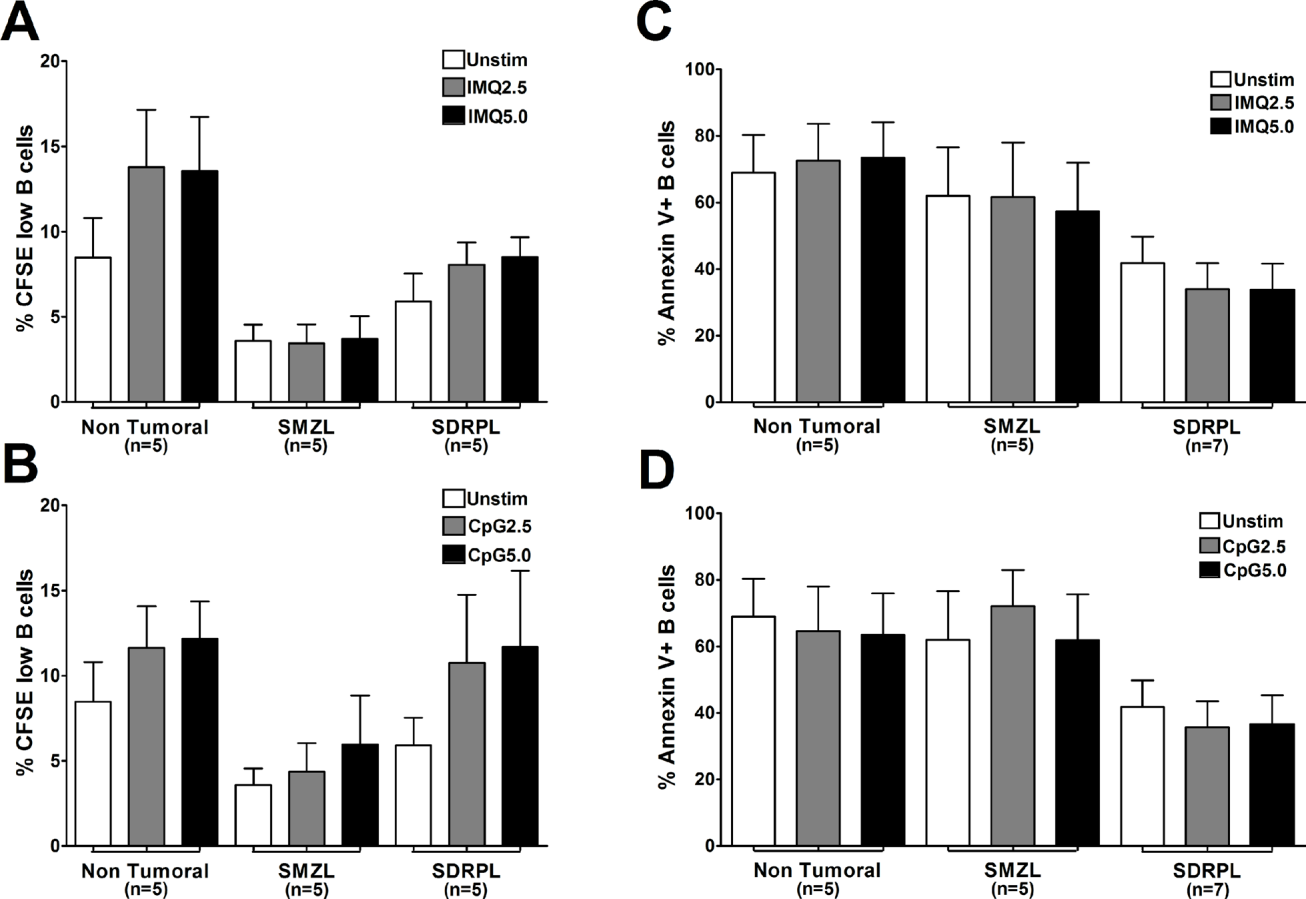
Proliferation and apoptosis upon TLR7 and TLR9 stimulations Proliferation of B cells in response to TLR stimulation was assessed by flow cytometry gated on living CFSE low CD19^+^ cells. TLR7 (IMQ) and TLR9 (CpG) agonists were both used at 2 concentrations (2.5 and 5 μg/mL) for 6 days. Results were expressed as mean ± SEM for each group (Non Tumoral, SMZL and SDRPL). (**A**) Percentage of circulating PB CFSE low B cells in response to unstimulated (Unstim) or TLR7 (IMQ) stimulation conditions. (**B**), Percentage of circulating PB CFSE low B cells in response to unstimulated (Unstim) or TLR9 (CpG) stimulation conditions Apoptosis of B cells in response to TLR stimulation was assessed by flow cytometry gated on Annexin V^+^ and CD19^+^ B cells. TLR7 (IMQ) and TLR9 (CpG) agonists were both used at 2 concentrations (2.5 and 5 μg/mL) for 48 hours. Results were expressed as mean ± SEM for each group (Non Tumoral, SMZL and SDRPL). (**C**) Percentage of circulating PB Annexin V^+^ B cells, in response to unstimulated (Unstim) or TLR7 (IMQ) stimulation conditions. (**D**) Percentage of circulating PB Annexin V^+^ B cells in response to unstimulated (Unstim) or TLR9 (CpG) stimulation conditions.

The potential impact of TLR stimulation on proliferation of circulating normal and tumoral B cells was then assessed by measuring the dilution of CFSE in living cells after 6 days of culture (Figure 3A, 3B). In both SMZL and SDRPL B cells, spontaneous proliferation was very low with less than 5% CFSE low cells. Stimulation with TLR7 and TLR9 agonists did not significantly increase proliferation in non-tumoral, SMZL or SDRPL circulating B cells (Figure 3A and 3B). We then assessed the impact of both TLR7 and TLR9 stimulations on apoptosis by measuring Annexin V positive B cells after 48 hours of culture. Constitutively, circulating SDRPL B cells were less apoptotic (41.85%), compared to SMZL B cells (62.02%), suggesting that circulating SDRPL B cells may be more resistant to apoptosis. Moreover, TLR7 and TLR9 stimulations did not significantly affect the proportion of apoptotic cells in SMZL and SDRPL B cells (Figure 3C, 3D).

### SDRPL and SMZL show different constitutive CD86 expression and expression of CD80 in response to TLR7 and TLR9 agonists

**Figure 4:**
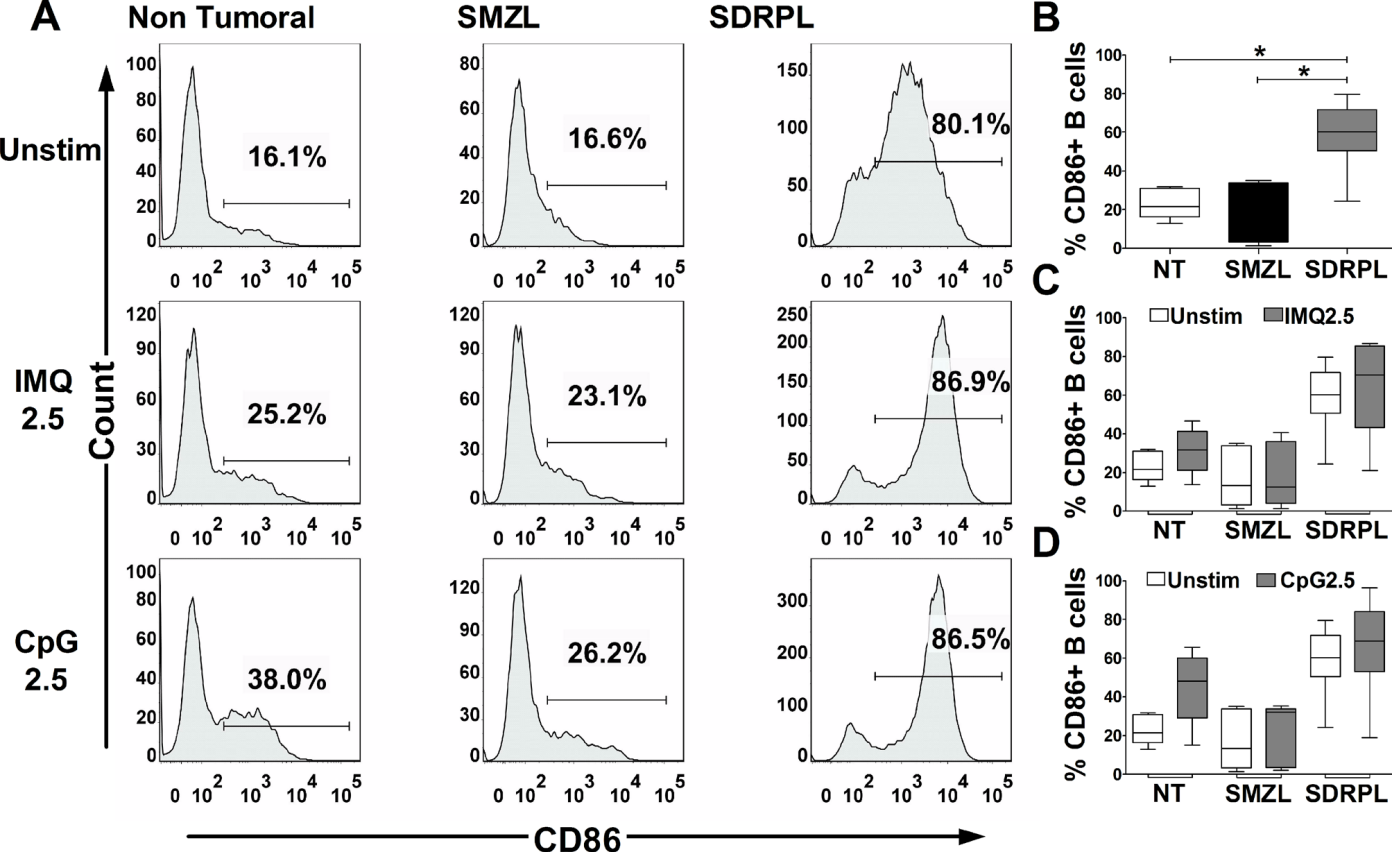
CD86 expression upon TLR7 and TLR9 stimulations CD86 expression was assessed by flow cytometry gated on CD19^+^ B cells. The TLR7 (IMQ) and TLR9 (CpG) stimulations were achieved with 2.5 μg/mL of each ligand for 24 hours. (**A**) Histograms of CD86 expression in circulating PB B cells (one demonstrative case of each group (*n* = 5): Non Tumoral, SMZL and SDRPL) under unstimulated (Unstim) or TLR stimulation conditions. (**B**) Percentage of CD86^+^ B cells in each group of circulating PB B cells (Non Tumoral (white box), SMZL (black box) and SDRPL (grey box)) in unstimulated conditions. (**C**) Percentage of circulating PB CD86^+^ B cells in unstimulated (white boxes) and TLR7 stimulated (grey boxes) conditions. (**D**) Percentage of circulating PB CD86^+^ B cells in unstimulated (white boxes) and TLR9 stimulated (grey boxes) conditions. Percentage of circulating PB CD86^+^ B cells are represented as box and whiskers (B–D). (^*^p < 0.05 and ^**^p < 0.01 with Mann-Whitney *t*-test).

**Figure 5:**
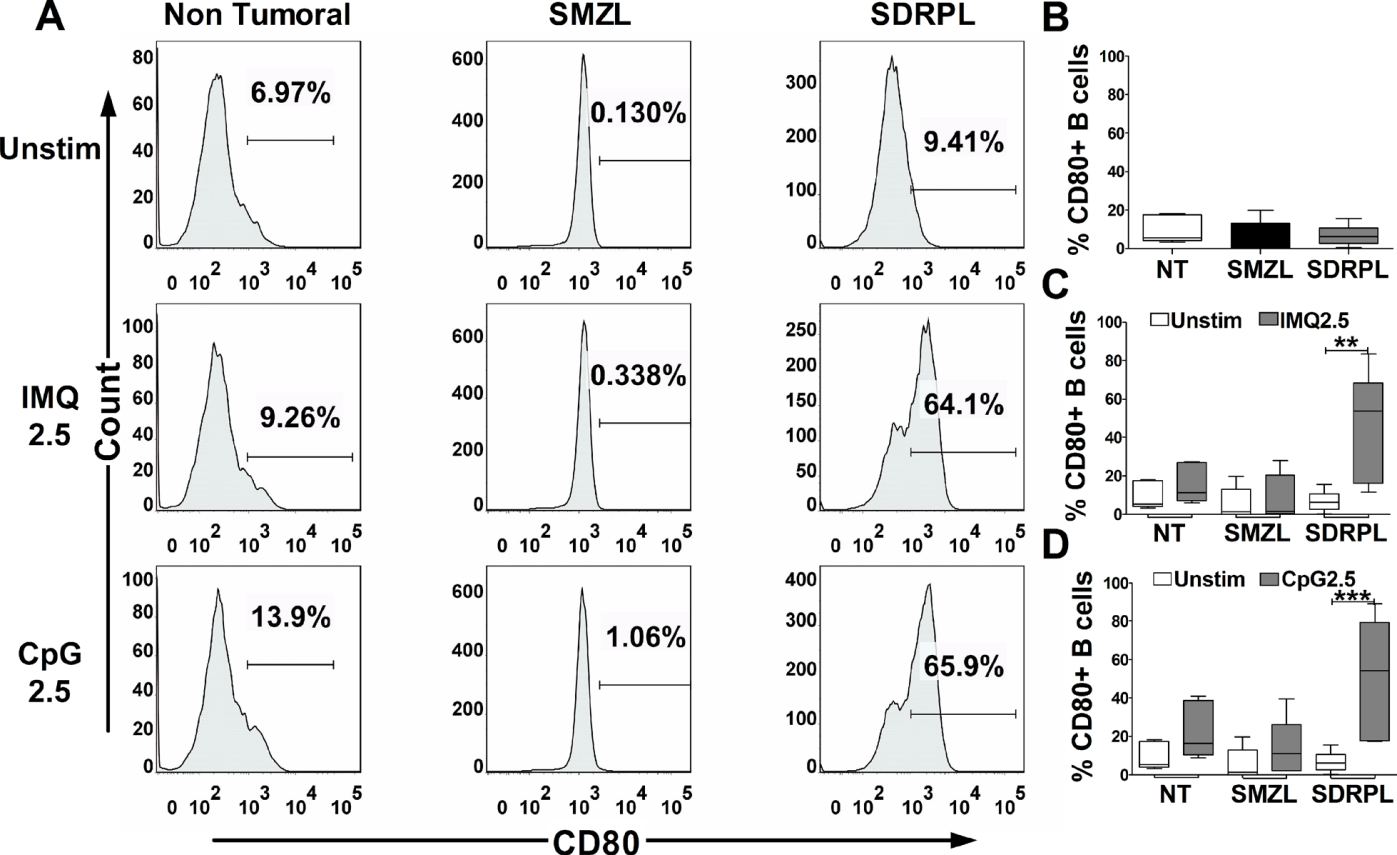
CD80 expression upon TLR7 and TLR9 stimulations CD80 expression was assessed by flow cytometry gated on CD19^+^ B cells. The TLR7 (IMQ) and TLR9 (CpG) stimulations were achieved with 2.5 μg/mL of each ligand for 24 hours. (**A**) Histograms of CD80 expression in circulating PB B cells (one demonstrative case of each group (*n* = 5): Non Tumoral, SMZL and SDRPL) under unstimulated (Unstim) or TLR stimulation conditions. (**B**) Percentage of CD80^+^ B cells in each group of circulating PB B cells (Non Tumoral (white box), SMZL (black box) and SDRPL (grey box)) in unstimulated conditions. (**C**) Percentage of circulating PB CD80^+^ B cells in unstimulated (white boxes) and TLR7 stimulated (grey boxes) conditions. (**D**) Percentage of circulating PB CD80^+^ B cells in unstimulated (white boxes) and TLR9 stimulated (grey boxes) conditions. Percentage of circulating PB CD80^+^ B cells are represented as box and whiskers (B–D). (^*^p < 0.05 and ^**^p < 0.01 with Mann-Whitney *t*-test).

The functional impact of TLR stimulation on B cell activation was next studied by FCM analysis of CD86/CD80 expression on circulating normal and tumoral B cells, after 24 hours of exposure to TLR7 and TLR9 agonists. Interestingly, circulating SDRPL B cells, but not SMZL B cells had a constitutive expression of CD86 (Figure 4A and 4B) (*p* < 0.05). TLR7 and TLR9 stimulations only moderately increased CD86 expression in non-tumoral, SMZL and SDRPL B cells (Figure 4A, 4C and 4D). On the other hand, SDRPL B cells did not constitutively express CD80 (Figure 5A and 5B) but highly expressed this marker after stimulation with both TLR7 and TLR9 agonists (Figure 5A, 5C and 5D) (*p* < 0.05 and *p* < 0.01 respectively). In contrast, TLR7 and TLR9 stimulations only induced a moderate increase in CD80 expression in non-tumoral and SMZL B cells (Figure 5A, 5C and 5D).

As TLR7 and TLR9 pathways were functional in splenic SMZL and SDRPL B cells, with an increased IL-6 secretion in response to the agonists (data not shown), we also analyzed CD86 and CD80 expression by those tumoral cells of splenic origin. Of note, in 2 SDRPL cases, splenic and circulating B cells obtained from the same patient were analyzed in parallel. Despite few samples studied, splenic SDRPL B cells constitutively expressed low level of CD86, compared to their circulating counterpart, but higher level compared to splenic SMZL B cells (Supplementary Figure 1A–1C). As reported for circulating SMZL and SDRPL B cells, CD80 was not constitutively expressed in their splenic form (Supplementary Figure 1D) but more surprisingly its expression was not increased after TLR7 or TLR9 stimulation in splenic SDRPL B cells (Supplementary Figure 1E and 1F).

## DISCUSSION

The present study focused on the TLR profile and function in two close splenic lymphomas entities: SMZL and SDRPL. To our knowledge and considering the rare incidence of the SDRPL, no data about TLR were nowadays reported in the literature and in particular no studies in both compartment in where tumoral SDRPL B cells can be detected. At the mRNA level and in splenic samples, we confirmed the higher expression of TLR9 in SMZL B cells compared to non-tumoral B cells, as previously reported [12], and described for the first time a TLR profile in SDRPL B cells. The TLR profiles were then analyzed by FCM on splenic and circulating B cells, showing the high expression of TLR7 and to a lesser extend the increased TLR9 in SDRPL. However, a few differences (in particular, lower TLR9 RFI than TLR7 RFI) were noted in comparison to previous profile reported by Fonte *et al.* in SMZL B cells [12]. These differences could be explained by the different presentation of the results (ie, normalized mean fluorescence intensity (RFI) of TLR expression among CD19^+^ SMZL B cells (in the present study) vs percentage of TLR-positive cells among CD19^+^ SMZL B cells in Fonte *et al.* study). We prefer to report TLR expression as RFI, since it gives supplementary information, such as the expression level.

Besides the distinct expression profiles of TLR between entities, SMZL and SDRPL B cells secreted IL-6 in response to the TLR7 agonist, confirming that TRL7 signaling is functional in those cells. In contrast, TLR9 signaling appears functional in SMZL B cells with high amount of IL-6 secreted in response to TLR9 agonist, whereas this response was weaker in SDRPL B cells. Therefore, in comparison to SMZL, circulating SDRPL B cells appear more sensitive to TLR7 than TLR9 stimulation.

Interestingly, no modification of proliferation or apoptosis was detected in our study in response to TLR7 and TLR9 stimulations, in both SMZL and SDRPL samples. Fonte *et al.* described an increased proliferation of SMZL circulating cells in response to TLR9 stimulation but the method was slightly different with Ki67 staining after 48 h [12], compared to a CFSE staining at 6 days in our study.

Expression of activation markers such as CD86 and CD80 already studied in response to TLR stimulation, appears different according to entities of other small B cells lymphomas. Indeed, an increased expression of both CD86 and CD80 in response to TLR9 stimulation was shown in chronic lymphocytic leukemia, but not in response to TLR7 stimulations [21]. Follicular lymphoma or mantle cell lymphoma also showed an increased expression of CD80 and/or CD86 in response to TLR9 agonist [10]. Nevertheless, B cells from nodal marginal zone lymphomas did not expressed those markers in response to TLR9 stimulation [10]. In the same manner, we showed that SMZL did not expressed CD86 or CD80 in response to TLR7 and TRL9 agonists. Interestingly, Fonte *et al.* showed an increased CD86 expression in response to TLR9 stimulation, but only in SMZL patients with mutated heavy chain immunoglobulin (IGHV) genes. Whether this different phenotype could be explained by mutational status of IGHV in our series remained to be explored.

We also reported for the first time the constitutive expression of CD86 in circulating and to a lesser extend splenic SDRPL B cells, compared to normal and SMZL B cells. Moreover, in response to TLR7 and TLR9 agonists, circulating SDRPL B cells show a drastic increase of CD80 expression. Interestingly the increased expression of CD80 after TLR stimulation was not found on the splenic counterpart of the SDRPL. Our recent NGS data [16] have reported an identical mutational profile in circulating and splenic SDRPL B cells, suggesting a same cell of origin between these two compartments. In addition, we analyzed the functional impact of TLR7 and TLR9 stimulations on 2 SDRPL samples with paired splenic and circulating B cells, confirming the difference of phenotype depending on the compartment studied. The present results may suggest that the microenvironment could impact the constitutive expression of activation markers such as CD86 and the potentiality of splenic SDRPL tumoral cells to respond to innate stimuli. Further studies should be done to precise the modulation of this phenotype according to the compartment, as it has consequences on future functional studies on SDRPL.

Moreover, the cytology of circulating tumoral B cells is very different between both entities. Indeed, SDRPL B cells are villous lymphocytes that correspond to lymphoid cells with characteristic cytoplasmic expansions [13]. Therefore in addition to their specific villous morphology, the high expression of CD86 (constitutively) and CD80 in response to TLR7 or TLR9 stimulation, suggests that circulating SDRPL B cells could be considered as professional antigen presenting cells. Whether this particular phenotype is directly responsible for the cytology and lymphomagenesis of SDRPL is still not known, but definitely deserves further investigation. Furthermore, the constitutive and high CD86 expression may be used as a new immunological criteria in the differential diagnosis between circulating SDRPL and SMZL and merit to be further evaluated in larger series.

In conclusion, circulating SMZL and SDRPL B cells may derive from different splenic B cells with specific immunological features (constitutive CD86 expression in SDRPL) that can be used as new and complementary immunological diagnosis markers contributing to its differential diagnosis with SMZL.

## MATERIALS AND METHODS

### Samples and cell purification

This study was performed on frozen peripheral blood (PB) and spleen cell suspensions from patients with SMZL (PB *n* = 5, spleen *n* = 15) and SDRPL (PB *n* = 7, spleen *n* = 6) diagnosed according to the 2008 WHO recommendations [22, 23]. The morphology, flow cytometry immunophenotyping, cytogenetics and molecular data were collected. Non-tumoral spleen samples (*n* = 7) and PB from healthy donors (*n* = 5) were used as controls. For functional analysis, the repartition of samples was as follows: non-tumoral (PB *n* = 5); SMZL (PB *n* = 5, spleen *n* = 2); SDRPL (PB *n* = 7, spleen *n* = 3, with 2 paired samples). Informed consent was obtained from all patients according to the Declaration of Helsinki.

All B cells from splenic and PB samples were negatively sorted with CD2 and CD14 antibodies by using magnetic separation (Miltenyi Biotec). The purity (>95%) of the B cell suspensions was assessed by flow cytometry after staining with an anti-CD19 antibody (clone HIB19, Becton Dickinson) and all B cells from lymphoma samples were monotypic without detection of residual polytypic B-cells. All the cell suspensions contained less than 1% of monocytes determined by flow cytometry after staining with an anti-CD14 antibody (clone M5E2).

### TLR and MyD88 gene and protein expressions

Expression levels of the 10 TLR, MyD88 and 5 endogenous genes were determined on cDNA from sorted B cells from splenic samples (purity >95%), using Taqman assays (Thermo Fisher Scientific). The Ct >35 were considered beyond the limit of detection. Relative expression was determined with the 2^−ΔCt^ method, where the geometric mean Ct of ACTB (Hs99999903_m1), TBP (Hs00427621_m1), GAPDH (Hs99999905_m1), RPL13A (Hs01926559_g1) and RPLP0 (Hs99999902_m1) was used as endogenous control [24]. The assays of the TLR and Myd88 are as follows: TLR1 (Hs00413978_m1), TLR2 (Hs00152932_m1), TLR3 (Hs01551078_m1), TLR4 (Hs00152929_m1), TLR5 (Hs01019558_m1), TLR6 (Hs00271977_s1), TLR7 (Hs00152971_m1), TLR8 (Hs00152972_m1), TLR9 (Hs00370913_s1), TLR10 (Hs01675179_m1) and Myd88 (Hs00182082_m1).

TLR protein expression gated on CD19^+^ cells from splenic and PB samples was acquired on FACS Canto II cytometer and analyzed using DIVA software (BD Biosciences) as previously described [14]. The expression of intracellular receptors: TLR3 (clone TLR3.7), TLR7 (clone 66H3), TLR8 (clone 307D3.01) and TLR9 (clone eB72-1665) were determined using the IntraPrep Permeabilization Reagent kit following manufacturer's recommendations (Beckman-Coulter). The surface receptors: TLR1 (clone GD2.F4), TLR2 (clone TL2.1), TLR4 (clone HTA125), TLR5 (clone 19D759.2), TLR6 (clone 86B1153.2) and TLR10 (clone 3C10C5) were determined as previously described [14]. The MyD88 expression (clone 603E10.05) was performed with the PerFix-no centrifuge assay (Beckman-Coulter,) on NAVIOS cytometer using NAVIOS software (Beckman-Coulter) on CD19^+^ gated cells.

The fluorescence levels for the different parameters were measured in arbitrary units, such as the mean fluorescence intensity (MFI) and the ratio of fluorescence intensity (RFI), which corresponds to the normalized MFI over the MFI of the negative control isotype.

### Cell culture and functional studies

For all functional assays, B cells from PB were purified (>95%) as described above. For the proliferation assay, these purified B cells were loaded with 0.25 μM of CFSE. TLR stimulation was performed with Imiquimod (IMQ) (Invivogen) for TLR7 or CpG: ODN 2006-G5 (CpG) (Invivogen) for TLR9 at final concentrations of 2.5 and 5 μg/mL. Cells were cultured in RPMI1640 medium supplemented with 10% FBS, 2 mM L-glutamine, 100 U/mL Penicillin and 100 mg/mL Streptomycin.

IL-6 production was measured with the DuoSet Human IL-6 kit (R&D Systems) from culture supernatants collected after 24 hours of culture in the presence or absence of IMQ or CpG.

All cytometry analysis were performed on LSRII cytometer (BD Biosciences) and analyzed with FlowJo software (FlowJo LLC). The expression of B cell activation markers CD80 (clone L307.4) and CD86 (clone IT2.2) was determined after 24 hours of culture. The percent of viable and apoptotic cells was analyzed using the Annexin V Apoptosis Detection Kit (Ebioscience) according to the manufacturer's instructions, after 2 days in culture. Proliferative cells (CFSE low) were determined in CD19^+^ cells that were negative for propidium iodide staining.

### Statistics

Comparison of expression levels of mRNA and RFI of TLR between lymphoma groups were determined by two-way ANOVA followed with Bonferroni test. Comparison of the different groups within splenic or PB samples were determined using a Mann-Whitney independent *t*-test (Graph Pad Software). *P*-values < 0.05 were considered significant.

## SUPPLEMENTARY MATERIALS FIGURES AND TABLES



## Abbreviations

BCR: B cell Receptor
CLL: Chronic Lymphocytic Leukemia
FCM: Flow Cytometry
IMQ: Imiquimod
MAPK: Mitogen Activated Protein Kinase
MFI: Mean Fluorescence Intensity
NF-κB: Nuclear Factor Kappa B
NGS: Next Generation Sequencing
PB: Peripheral Blood
RFI: Relative Fluorescence Intensity
SDRPL: Splenic Diffuse Red Pulp Lymphoma
SMZL: Splenic Marginal Zone Lymphoma
TLR: Toll-like Receptor
