# Supplementary material for: Toll-like receptor expression and function differ between splenic marginal zone B cell lymphoma and splenic diffuse red pulp B cell lymphoma
Source: Oncotarget. 2018 May 4;9(34):23589–98. doi: 10.18632/oncotarget.25283 (PMC5955093; doi:10.18632/oncotarget.25283)
Supplement: Supplementary file 1 [file oncotarget-09-23589-s001.pdf]

Toll-like receptor expression and function differ between splenic marginal zone B cell lymphoma and splenic diffuse red pulp B cell lymphoma

SUPPLEMENTARY MATERIALS

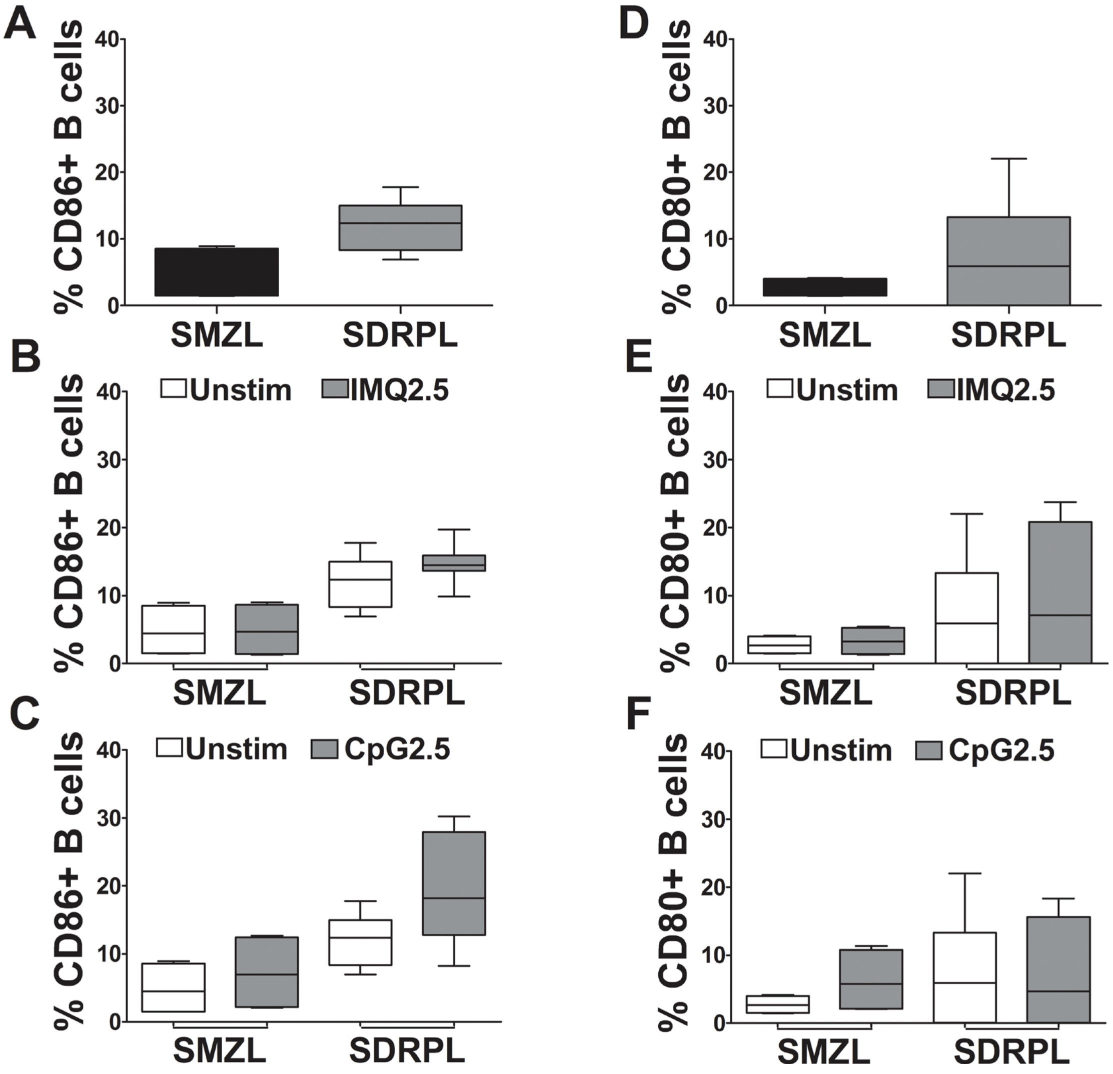

Supplementary Figure 1: CD86 and CD80 expression upon TLR7 and TLR9 stimulations on splenic B cells. CD86 and CD80 expressions were assessed by flow cytometry gated on CD19$^+$ B cells. The TLR7 (IMQ) and TLR9 (CpG) stimulations were achieved with 2.5 µg/mL of each ligand for 24 hours (**A**) Percentage of CD86$^+$ B cells in each group of splenic B cells, SMZL (black box) and SDRPL (grey box) in unstimulated conditions. (**B**) Percentage of splenic CD86$^+$ B cells in unstimulated (white boxes) and TLR7 stimulated (grey boxes) conditions. (**C**) Percentage of circulating PB CD86$^+$ B cells in unstimulated (white boxes) and TLR9 stimulated (grey boxes) conditions. (**D**) Percentage of CD80$^+$ B cells in each group of splenic B cells, SMZL (black box) and SDRPL (grey box) in unstimulated conditions. (**E**) Percentage of splenic CD80$^+$ B cells in unstimulated (white boxes) and TLR7 stimulated (grey boxes) conditions. (**F**) Percentage of circulating PB CD80$^+$ B cells in unstimulated (white boxes) and TLR9 stimulated (grey boxes) conditions. Percentage of splenic CD86$^+$ and CD80$^+$ B cells are represented as box and whiskers with SMZL, $n = 2$ and SDRPL, $n = 3$.
